# Computational analysis of CCN1 as a druggable target predicts interactions with bioactive compounds

**DOI:** 10.1038/s41598-025-34139-4

**Published:** 2026-01-13

**Authors:** Roudy Bou Francis, Racha Kerek, Mohamad Rima

**Affiliations:** Department of Biological Sciences, Lebanese American University, Byblos, Lebanon

**Keywords:** CCN1, AlphaFold 3, SwissDock, Fpocket, Druggability, *In silico*, Computational biology and bioinformatics, Drug discovery

## Abstract

*In silico* druggability assessment helps shorten early drug discovery by identifying small molecules worth experimental testing as potential protein modulators. CCN1 is a multifunctional protein involved in various physiological processes and its dysregulation has been implicated in pathological conditions such as aging, fibrosis, inflammation, and cancer. The diverse, and sometimes contradictory, functions of CCN1 make it an important candidate for druggability assessment. In this study, we evaluated its druggability by predicting its 3D structure using AlphaFold 3, identifying binding pockets with Fpocket, and assessing ligand affinity with SwissDock. Our integrative *in silico* workflow identified multiple high-confidence druggable pockets within the CCN1 protein, with the top-scoring site located between the thrombospondin type 1 (TSP-1) and C-terminal cystine knot (CTCK) domains. Molecular docking predicted strong interactions with several clinically relevant compounds, including antioxidants and senolytics, with Metformin showing the highest affinity (SwissDock AC score: -200.26). Importantly, these ligand-binding interactions remained stable even after deletion of amino acids forming the predicted pocket and across naturally occurring CCN1 variants arising from SNPs, indicating that CCN1 is a genetically robust drug target. This study is the first to computationally demonstrate the druggability of CCN1 and to identify candidate small molecules with the potential to modulate its activity in aging- and disease-related contexts. Our findings provide both mechanistic insight and a scalable workflow for rapid screening of CCN1-targeted therapeutics.

**Supplementary Information:**

The online version contains supplementary material available at 10.1038/s41598-025-34139-4.

## Introduction

CCN1, also known as CYR61 (Cysteine-rich angiogenic inducer 61), is a secreted matricellular protein and a founding member of the CCN family^1^, named after its first identified members: CCN1 (CYR61), CCN2 (CTGF or Connective tissue growth factor), and CCN3 (NOV or Nephroblastoma overexpressed). CCN1 is composed of four conserved domains: an insulin-like growth factor binding protein (IGFBP)-like domain, a von Willebrand factor type C (VWFC) repeat, a thrombospondin type 1 repeat (TSP-1), and a C-terminal (CT) cystine knot (CTCK) domain^2,3^. These domains are separated by flexible linker regions and collectively mediate CCN1’s interactions with a wide array of receptors and ligands. The TSP-1 and CTCK domains, in particular, are critical for binding integrins, Wnt and heparan sulfate proteoglycans, thus enabling CCN1 to anchor to the extracellular matrix (ECM) and transmit signals across the plasma membrane^4,5^. Through this dynamic mediation of cell-ECM interactions, CCN1 triggers downstream signaling pathways that regulate multiple context-depending cellular processes such adhesion, migration, proliferation, differentiation, and apoptosis^1,6^. Therefore, CCN1 was classified as a modular protein that plays a crucial role in different physiological processes including angiogenesis, wound healing, tissue regeneration, embryonic development, and cellular senescence^3^.

In healthy tissues, CCN1 helps orchestrate the balance between tissue repair and remodeling. However, dysregulation of CCN1 expression or activity has been linked to a wide spectrum of pathological conditions, including fibrosis, inflammation, vascular disorders, aging-related degeneration, and cancer^1,7,8^. These diverse tissue- and context-dependent functions of CCN1 underscores its importance as a therapeutic target. Therefore, several bioactive compounds have been identified for their ability to modulate CCN1-related pathways, yet direct interactions with CCN1 have not been systematically investigated^9,10^. However, there is an ongoing need for CCN1-targeting drugs tailored to specific disease contexts. Recent *in silico* studies have emphasized the importance of computational approaches in advancing research efforts targeting CCN1. For example, differential gene expression and network analyses have identified CCN1 as a potential therapeutic target in colorectal cancer for example^11^, while integrative machine learning studies have enhanced feature selection and phenotype classification, highlighting CCN1 among key discriminatory genes^12^. Despite the clear biological and clinical relevance of CCN1, its potential as a direct therapeutic target remains largely unexplored. The absence of a resolved crystal structure for CCN1^5^ has historically constrained mechanistic understanding. However, recent advances in deep learning-based structure prediction tools, such as AlphaFold 3, now provide high-confidence models that enable *in silico* structural and functional analyses as preparatory steps for experimental validation^13,14^. Before the advent of bioinformatic approaches, protein structure and druggability were evaluated exclusively through experimental methods. However, the development of *in silico* tools has since revolutionized and greatly accelerated the early stages of drug discovery.

The present study employs a computational approach to explore the druggability of CCN1 by assessing its interactions with selected natural compounds and commonly used drugs. Specifically, we predicted the 3D structure of CCN1 using AlphaFold 3, identified potential small-molecule binding pockets with Fpocket, and evaluated ligand-binding affinities using SwissDock, a flexible docking algorithm. Our goal was to determine whether CCN1 possesses structurally defined, ligand-accessible sites that could be targeted by pharmacologically active compounds. Additionally, we investigated whether CCN1 mutants alter binding potential, thereby assessing the robustness of ligand interactions across diverse genetic backgrounds. This study represents the first comprehensive computational exploration of natural compounds and small-molecule drugs with potential binding affinity to CCN1, a protein whose modulation could have therapeutic relevance across diverse conditions, including fibrosis, cancer, and aging.

## Materials and methods

### Protein structure prediction and validation

The three-dimensional structure of CCN1 was predicted using AlphaFold 3, a deep learning-based protein structure-prediction tool developed by DeepMind^14^ and hosted at https://deepmind.google/science/alphafold/. The amino acid sequence of human CCN1 was retrieved from the UniProt database (Accession: O00622) in FASTA format before submission to the AlphaFold 3 interface, which incorporates a unified architecture for proteins, ligands, nucleic acids, and post-translational modifications. Predictions were run using the default parameters. Prior to proceeding with any downstream analysis, the AlphaFold predicted CCN1 models were structurally assessed using pLDDT scores (predicted Local Distance Difference Test), with values above 70 considered to indicate reliable predictions. Additional structural validation was performed to assess the overall stereochemical quality of our model using PROCHECK^15,16^ implemented in the SAVES v6.1 Structure Validation Server (https://saves.mbi.ucla.edu). A Ramachandran plot was subsequently generated to evaluate the protein’s 3D structure by analyzing the backbone dihedral angles (φ and ψ) of all amino acid residues. This analysis determined the proportion of residues located within the most favored regions (typically corresponding to α-helices and β-sheets), as well as those in additionally allowed, generously allowed, or disallowed regions.

The predicted structures were downloaded and visualized using PyMOL (version 2.5.4) for downstream structural analyses. All predicted structures were exported to a web-based tool^17^ for conversion from mmCIF to PDB format, generating input files compatible with Fpocket. To model the Q238H (rs759768515) and T242A (rs1659812460) variants, as well as the multiple-point deletion mutant, the reference sequence was manually edited to reflect the amino acid substitutions or deletions. The modified sequences were then submitted to AlphaFold 3 using the same parameters as for the wild-type.

### Pocket prediction by fpocket

Potential ligand-binding pockets were predicted using Fpocket^17^, an open-source protein cavity detection tool based on Voronoi tessellation and alpha sphere geometry. The 3D structures of CCN1 predicted via AlphaFold 3 were used as input in PDB format, and Fpocket was run using default parameters. Fpocket generated a ranked list of predicted pockets based on volume, polarity, hydrophobicity, and druggability scores. The top-ranked pocket, exhibiting the highest druggability score, was selected for further analysis. Pocket visualization and residue mapping were performed using PyMOL (version 2.5.4). Wild-type and mutant/variants CCN1 structures were processed independently using the same pipeline and parameters to ensure consistency.

### Ligand selection

A set of small molecules was chosen for docking based on their known or hypothesized biological activity related to the functions of CCN1: aging, inflammation, cancer or metabolic regulation^3^. These included: Antioxidants (e.g., quercetin, resveratrol, epigallocatechin-3-gallate (EGCG)), senolytic agents (e.g., piperlongumine), chemotherapeutics (e.g., doxorubicin) and repurposed drugs with anti-aging or anti-fibrotic properties (e.g., metformin).

**Table 1:** List of drugs used for molecular docking (retrieved on January 4, 2025).

Drug/compound	PubChem CID	Drug/compound	PubChem CID
Pirfenidone	40632	Quercetin	5280343
Naringenin	439246	Fisetin	5281614
Vitamin C	155903693	Metformin	4091
Vitamin A (retinol)	445354	Carmustine	2578
Resveratrol	445154	Etoposide	36462
EGCG (green tea)	65064	Clonidine	2803
Glutathione	124886	Metoprolol	4171
Amlodipine	2162	Esmolol	59768
Piperlongumine	637858		

### Molecular docking

Molecular docking was performed using SwissDock (http://www.swissdock.ch/), based on the EADock DSS algorithm^18,19^. The CCN1 protein structure containing the most druggable pocket, as predicted by Fpocket, was used for docking simulations. Ligand structures were obtained from PubChem (Table 1) and uploaded as SMILES files. Docking was conducted in a targeted manner, focusing specifically on the CCN1 domain containing the pocket with the highest druggability score, which was predefined as the binding site. The docking parameters are summarized in Table 2, and results were ranked based on the Full Fitness score and the estimated binding free energy (ΔG), also referred to as the AC score, where lower AC scores indicate stronger binding affinities. High-quality visualizations of ligand-protein interactions were generated using BIOVIA Discovery Studio and hydrogen bonding, hydrophobic interactions, and ligand stability were analyzed using the software’s interaction analysis tools.

**Table 2:** SwissDock docking parameters. The box size and center were manually adjusted to better encompass the CCN1 domain harboring the most druggable pocket.

Target	_*wt*_CCN1	_*mutant*_CCN1	_*Q238H variant*_CCN1	_*T242A variant*_CCN1
Method	AC			
Sampling exhaustivity	90 (medium)			
Cavity prioritization	60 (medium)			
Random initial conditions	1			
Box size	30/47/37	29/34/21		
Box center	-25/-11/-25	-22/2/-9	-10/17/5	8/-19/7

## Results

### 3D structural analysis of CCN1

**Fig. 1:**
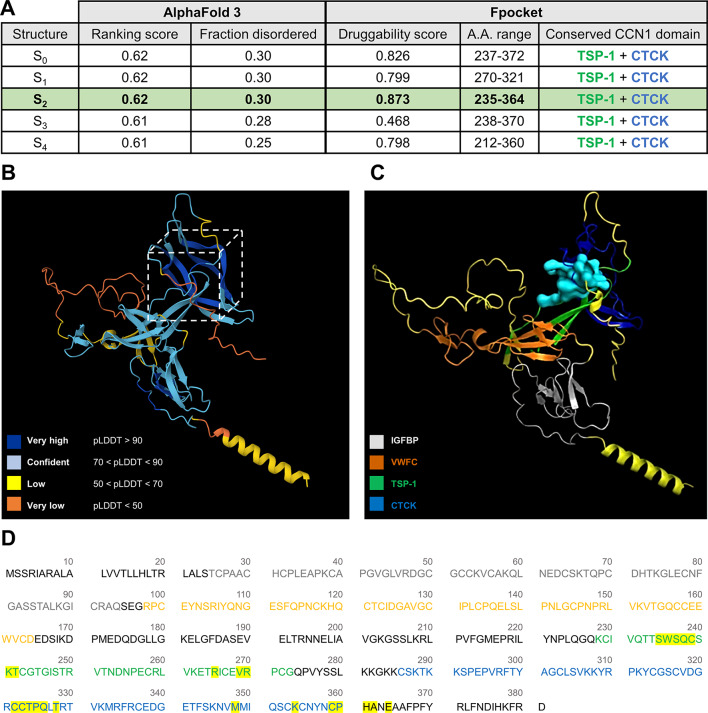
Exploration of CCN1 druggability of (**A**) AlphaFold 3 scores of the different predicted structures S_0_-S_4_. For each structure, druggable pockets were identified using Fpocket, and the amino acids forming the pocket with the highest druggability score were mapped to the corresponding CCN1 domains. Of note, only the pocket with the highest druggability score is represented for each structure. The detailed results of druggable pocket predictions using Fpocket are present in Supplementary Fig. 2. (**B**) 3D structure of CCN1 (S_2_) predicted by AlphaFold 3. The structure is colored by pLDDT (predicted Local Distance Difference Test) scores, reflecting the confidence of AlphaFold predictions. The dashed box highlights the region of interest holding the druggable pocket with the highest druggability score. The high-confidence regions suggest reliable structural predictions. (**C**) CCN1 structure (S_2_) predicted by AlphaFold 3 showing the chosen druggable pocket (in cyan). (**D**) CCN1 sequence showing its different domains and the a.a. forming the predicted druggable pocket of S_2_ highlighted in yellow. The different protein domains in (**C**) and (**D**) are color coded as follow: IGFBP in grey, VWFC in orange, TSP-1 in green, and CTCK in blue. The predicted pocket is nested mainly in the conserved TSP-1 and CTCK domains.

We began by predicting the 3D structure of CCN1 using AlphaFold 3^20,21^. The tool suggested five possible structures with slightly different but closely ranked confidence and fraction disorder scores (Fig. 1A), suggesting that CCN1 can adopt more than one stable or semi-stable conformation. This is consistent with the known architecture of CCN1, which comprises distinct and individually structured domains connected by flexible linkers^1^ that facilitate its dynamics and conformational switching, features characteristic of signaling proteins^2^. Ramachandran plot analysis using PROCHECK revealed that the predicted AlphaFold model had 84.0% of residues in the most favored regions (α-helices and β-sheets), 14.4% in additionally allowed regions, 0.9% in generously allowed regions, and only 0.6% in disallowed regions (Supplementary Fig. 1). The few potential outliers detected (i.e., GLU173, ASP176) are located within a surface-accessible loop predicted by AlphaFold to be flexible, with very low pLDDT confidence (< 50). These results indicate an overall robust and stereochemically reliable predicted structure.

### Prediction of druggable binding pockets in CCN1

Using the Fpocket online tool^22,23^, we investigated the presence of druggable pockets within the five CCN1 structural models predicted by AlphaFold 3. Multiple pockets were detected in each model. While some exhibited no druggability (druggability score = 0), others showed high druggability (druggability score > 0.7) (Supplementary Fig. 2A). The pockets with the highest druggability scores were: 0.826 for pocket #20 in structure S_0_, 0.799 for pocket #30 in structure S_1_, 0.873 for pocket #16 in structure S_2_, 0.468 for pocket #34 in structure S_3_, and 0.798 for pocket #6 in structure S_4_. For each structure, the predicted pocket with the highest druggability score was mapped to its position within the CCN1 sequence and structure. The amino acids (a.a.) forming these pockets were consistently located within the TSP-1 and CTCK domains (Fig. 1A), suggesting that highly druggable pockets are concentrated in the C-terminal domain of the protein. Of note, the TSP-1 and CTCK are functionally critical domains, mediating most CCN1 activities by binding and interacting with various receptors^3^. In particular, these domains regulate both integrin binding and Wnt/Frizzled pathway, two major pathways through which CCN1 modulates senescence, fibrosis, angiogenesis and stem cell regulation^3^. Notably, structure S_2_ contained the highest number of druggable pockets and the lowest number of non-druggable pockets among all predicted models (Supplementary Fig. 2B and C). Within S_2_, pocket #16 displayed the highest druggability score (druggability score = 0.873) across all predicted pockets in all the models (Fig. 1A, Supplementary Fig. 2A). Among the three top-scoring pockets identified in structure S_2_, this pocket exhibits the largest volume (828 Å³), a high α-sphere density (6.76), and a balanced polar/apolar ratio (~ 46% polar) (Supplementary Fig. 2D). In contrast, pockets 21 and 34 display moderate and large volumes, respectively, and are characterized by predominantly hydrophobic and hydrophilic environments (Supplementary Fig. 2D). The combination of large volume, optimal polarity balance, and high α-sphere density suggests that this pocket may represent the most favorable site for potential ligand binding.

Based on its high structural ranking score and highest druggable pocket score, indicating enhanced reliability, S_2_ was selected for further investigation. The 3D conformation of the protein, retrieved from AlphaFold 3, was visualized with the corresponding confidence scores (pLDDT) (Fig. 1B). Analysis revealed that pocket #16 resides within a region of very high structural confidence (pLDDT > 90), indicating a well-defined and reliable local conformation likely suitable for structure-based studies such as ligand docking or virtual screening (Fig. 1B). In PyMOL, this pocket was further visualized in cyan within the protein 3D conformation with the respective color-coded domains (Fig. 1C). The amino acids forming this pocket were also mapped onto the primary sequence, confirming their localization within the TSP-1 and CTCK domains (Fig. 1D).

###  Molecular docking simulation of CCN1-drug interaction

Next, we extended our analysis to investigate the druggability of the identified pocket in different CCN1-related disease contexts. Molecular docking simulations were performed using SwissDock^18,19^ to evaluate the binding potential between the TSP-1/CTCK domains of CCN1 and a panel of ligands with known relevance to CCN1-mediated functions, including antifibrotics, senolytics, antioxidants, antidiabetic/antisenescence, chemotherapeutic and antihypertensive drugs^3^.

**Fig. 2:**
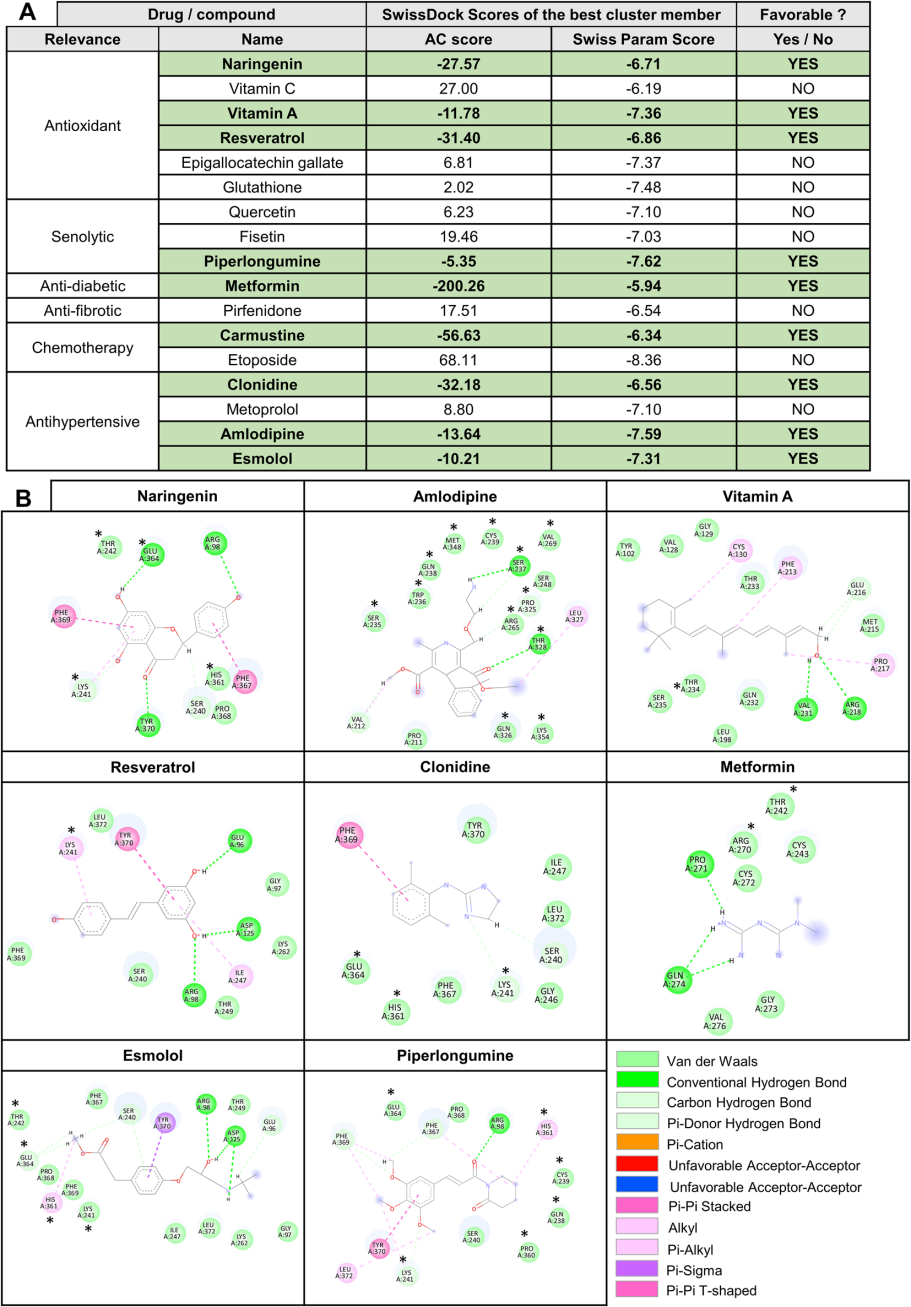
Molecular docking of CCN1 with different drugs/compounds. (**A**) List of selected ligands investigated for their interaction with the TSP-1/CTCK domains of CCN1 structure S_2_ using SwissDock. The key metric scores Affinity energy score (AC) and SwissParam scores are shown to predict the ligand-protein binding affinity. For each drug different clusters are predicted, only the one with the lowest AC score (favorable) is shown. (**B**) 2D representation of protein-ligand interactions between CCN1 TSP-1 and CTCK domains and the compounds showing a favorable affinity to CCN1. Grey “clouds” on ligand atoms indicate the solvent-exposed surface area. The amino acids marked with an asterisk (*) are those predicted to form the druggable pocket.

Docking analyses revealed a cluster of predicted interactions between CCN1 and several compounds (Fig. 2A). Binding affinity was assessed using the Affinity Energy (AC) and SwissParam scores generated by SwissDock, key metric used to predict ligand-protein binding strength, where a lower (more negative) value reflects a stronger and more favorable binding affinity of the ligand-protein complex^18^. Interestingly, antioxidant molecules such as naringenin, vitamin A (retinol), and resveratrol are predicted to interact with CCN1 at different affinities as seen with their corresponding low AC and SwissParam scores (Fig. 2A). In contrast, other antioxidants, such as vitamin C, epigallocatechin gallate, and glutathione, showed positive AC scores, suggesting no binding affinity to CCN1 *via* its TSP-1/CTCK domains.

Given CCN1’s important role in senescence^3^, we also assessed its possible interaction with senolytic drugs. Among the three tested senolytics, piperlongumine was the only compound showing favorable interaction with favorable (negative) AC scores. Metformin, an antioxidant, antidiabetic, and anti-senescence drug, displayed the lowest AC and SwissParam scores among all tested compounds, suggesting a particularly a favorable and potential strong binding interaction with CCN1 (Fig. 2A). Among antifibrotic agents, pirfenidone was not predicted to interact with the druggable pocket; however, the chemotherapeutic carmustine exhibited potential binding interaction (Fig. 2A), whereas etoposide showed no interaction, with CCN1. Regarding antihypertensive drugs, three of the four tested compounds, clonidine, amlodipine, and esmolol, were predicted to bind the CCN1 druggable pocket, while only metoprolol showed no binding affinity (Fig. 2A). Taken together, our findings identify a set of candidate compounds with the potential to interact with CCN1, offering novel insights into its druggability across diverse disease contexts. This list is not exhaustive and can be updated with additional drugs and pharmacologically relevant molecules.

### Visualization of CCN1-drug interactions

BIOVIA discovery studio was used to visualize the amino acids involved in the interactions with the favorably binding ligands. Interestingly, some ligands showed direct interactions with amino acids forming the druggable pocket, whereas others are predicted to interact with residues nested within the druggable pocket (Fig. 2B). For example, naringenin is predicted to interact with 10 amino acids, 4 of which form the pocket (Fig. 2B), while amlodipine interacts with 16 amino acids, 12 of which are part of the pocket. These interactions are also diverse in nature, involving Van der Waals forces, hydrogen bonds, π–π interactions, and others (Fig. 2B). Such findings highlight structural diversity in the binding modes of candidate compounds and raises questions of whether mutations affecting these amino acids could impair candidate drug binding.

**Fig. 3:**
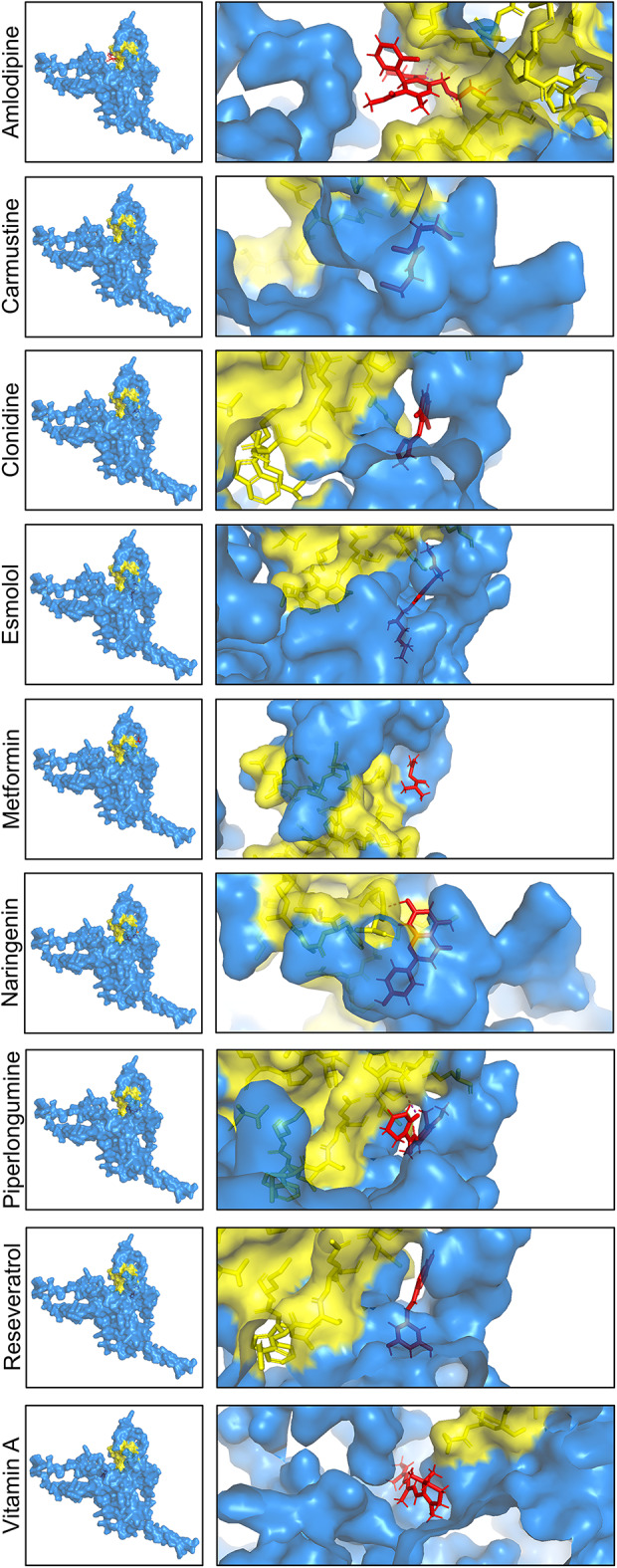
Surface representation of CCN1 with docked small molecules. The 3D structure of CCN1 is shown as a surface in blue. The predicted druggable pocket is highlighted in yellow, while the docked drug is shown in red. The zoomed-in views in each panel illustrate the binding proximity and orientation of selected compounds. These varied binding poses highlight the structural flexibility of the CCN1 pocket and its ability to accommodate ligands through distinct interaction modes.

To further visualize CCN1-drug binding interactions, molecular docking results were rendered in PyMOL (Fig. 3). This figure illustrates the nine small molecules identified as potential CCN1 interactors, occupying a defined surface cavity. The protein surface is shown in blue, with the predicted binding pocket highlighted in yellow and the docked drug in red; a zoomed-in view is also provided in each panel. All nine compounds exhibited binding within relatively similar or adjacent regions, supporting the presence of a conserved druggable site on CCN1. A zoomed-in view suggested the potential binding positions of the ligands within the CCN1 3D structure. Amlodipine was located in close proximity to the CCN1 druggable pocket, while carmustine and clonidine were positioned adjacent to this region (Fig. 3). Metformin engages near the external rim of the pocket, without being embedded into the pocket core. These differences suggest that the CCN1 druggable pocket is structurally versatile, accommodating a range of ligands with distinct sizes, shapes and chemical properties through distinct binding modes. While some compounds, such as amlodipine engage deeply within the pocket, others such as clonidine and metformin interact more peripherally or transiently. This versatility highlights the potential for targeting CCN1 with a structurally diverse range of small molecules.

### CCN1-drug favorable interactions are maintained after sequence alterations of CCN1

**Fig. 4:**
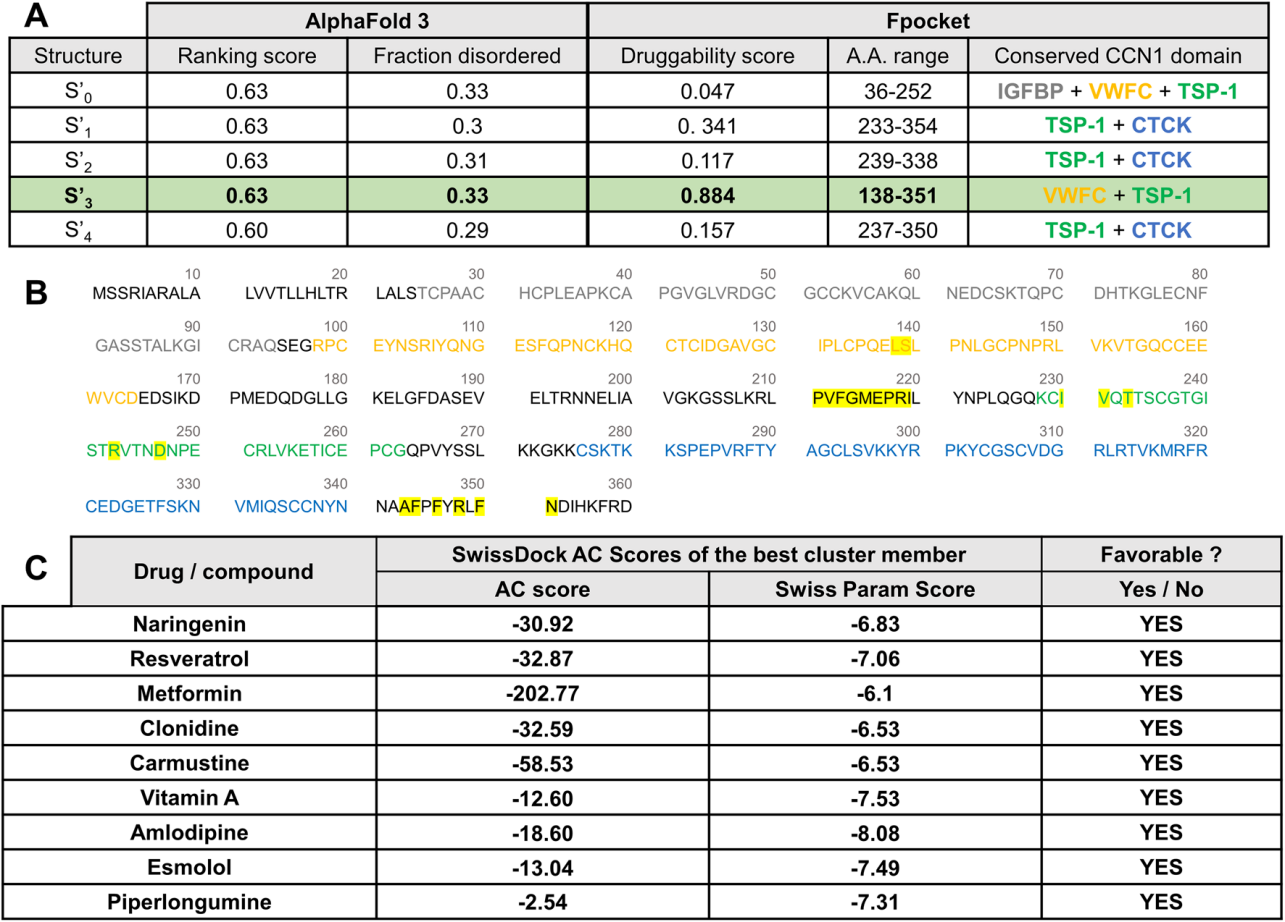
Stability of CCN1-ligand interactions after the alteration of the CCN1 sequence. (**A**) AlphaFold 3 scores of the different predicted structures of CCN1 (S’_0_-S’_4_) after deleting all the a.a. forming the highest druggable pocket selected in Fig. 1. For each structure, the presence of druggable pockets was investigated using Fpocket and the a.a. forming the pocket with the highest druggability score were positioned in CCN1 domains. Of note, only the pocket with the highest druggability score is represented for each structure. (**B**) _*mutant*_CCN1 sequence showing its different domains and the a.a. forming the highest predicted druggable pocket in S’_3_ highlighted in yellow. The different protein domains are color coded as follow: IGFBP in grey, VWFC in orange, TSP-1 in green, and CTCK in blue. The predicted pocket is mainly nested in the conserved TSP-1 domains, with some a.a. spanning the flexible linker and CT domain shown in black. (**C**) Selected compounds predicted to bind wild-type CCN1 (_*wt*_CCN1) were analyzed for their interaction with the mutant CCN1 structure S’_3_ using SwissDock. The key metric scores Affinity energy score (AC) and SwissParam scores are shown to predict the ligand-protein binding affinity. For each compound different clusters are predicted, only the one with the lowest AC score (favorable) is shown.

To further assess the stability of previously predicted CCN1-drug interactions, we deleted the amino acids that were identified by Fpocket as the ones forming the most druggable binding pocket. This artificial multiple-point deletion was introduced into the protein sequence to investigate the structural and functional consequences of removing key residues presumed to be critical for pocket stability, folding, and interaction with these compounds. The 3D structure of the truncated CCN1 was then retrieved using AlphaFold 3, generating five different possible structures (S’_0_-S’_4_) with close ranking and fraction disorder scores (Fig. 4A). Fpocket analysis revealed that the most druggable pocket in these mutant structures was predicted in S’_3_ (Fig. 4A), which showed a moderate folding change compared to wild-type CCN1 (_*wt*_CCN1) (Supplementary Fig. 3). By looking into the position of the amino acids forming the druggable pocket in S’_3_, they were primarily located in the VWFC and TSP-1 domains, with some also present in the flexible linkers (Fig. 4B). Ramachandran plot analysis revealed that the predicted AlphaFold model had 81.9% of residues in the most favored regions (α-helices and β-sheets), 15.8% in additionally allowed regions, 2% in generously allowed regions, and only 0.3% in disallowed regions (Supplementary Fig. 1). The potential outlier detected (ASN195) is located within a surface-accessible loop predicted by AlphaFold to be flexible. Notably, these structural and conformational changes did not abolish the affinity of the multiple-point deletion mutant (_*mutant*_CCN1) for ligands predicted to bind to the _*wt*_CCN1 (naringenin, resveratrol, metformin, clonidine, carmustine, vitamin A, amlodipine, esmolol, and piperlongumine) (Fig. 4C). Interestingly, all these compounds except piperlongumine exhibited slight decrease in AC scores suggesting increased affinity to _*mutant*_CCN1 (Figs. 2A and 4C).

**Fig. 5:**
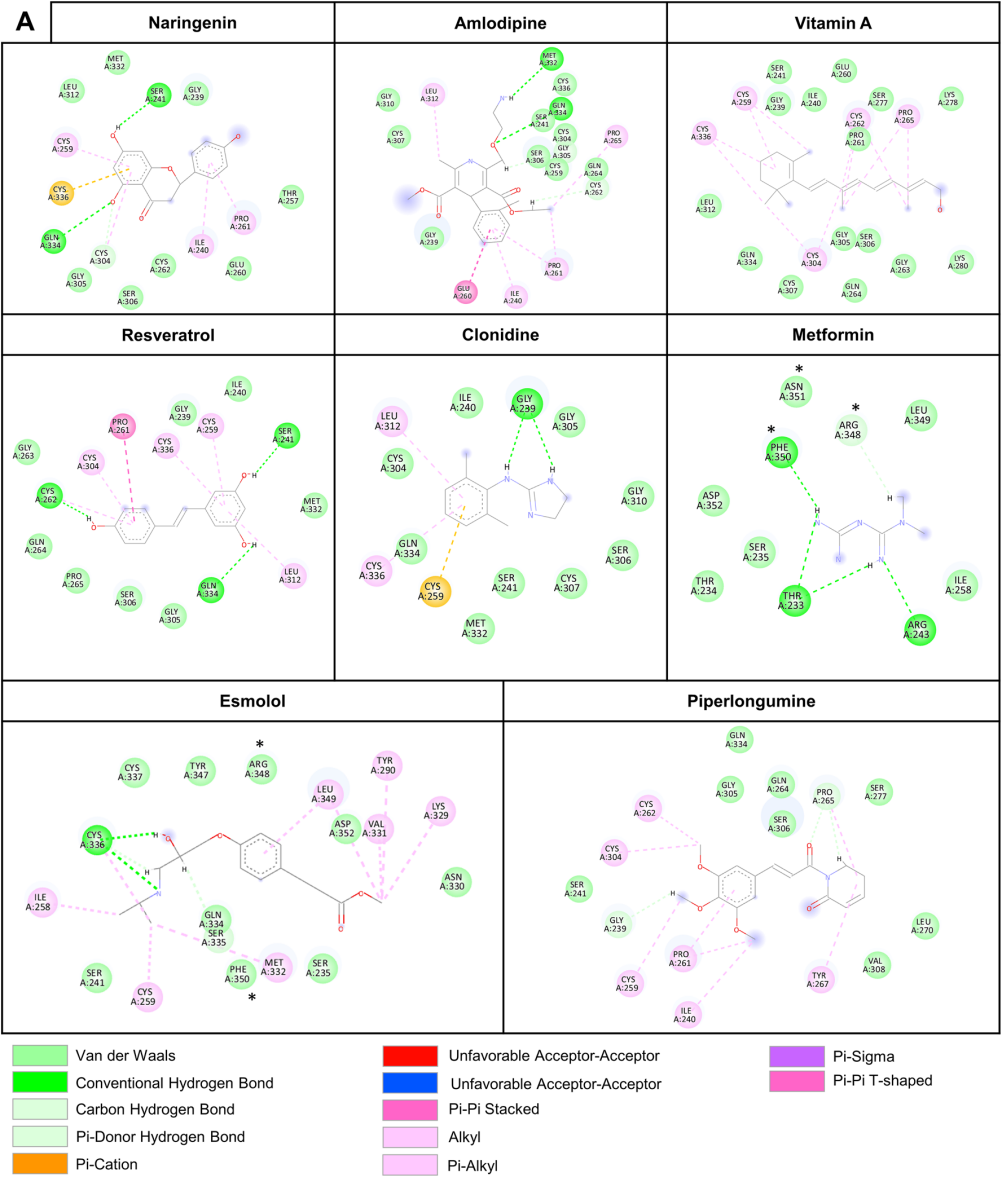
In-depth analysis of the predicted interactions between favorable ligands and the druggable pocket of _*mutant*_CCN1. Shown is a 2D representation of protein-ligand interactions involving the TSP-1 domain of _*mutant*_CCN1 structure S’_3_ and ligands with favorable predicted affinity. Gray “clouds” on ligand atoms represent solvent-exposed surface areas. Amino acids marked with an asterisk (*) correspond to residues predicted to form the druggable pocket.

Of interest, the compounds retained binding capacity with the mutant protein despite the deletion of key amino acids forming the most druggable pocket in _*wt*_CCN1. This may be explained by the emergence of new interactions between the compounds and _*mutant*_CCN1 that may be stronger than those predicted with _*wt*_CCN1 (Fig. 5). For instance, the improved binding affinity in amlodipine-CCN1 complex could be attributed to the addition of amide–π stacking and a denser network of π-alkyl, alkyl, and hydrogen bond interactions, enhancing both stability and molecular complementarity (Fig. 5). Metformin binding also remained highly favorable after deletion, with hydrogen bonding preserved and redistributed. Its small size and high polarity may enable flexible adaptation to altered binding environments (Fig. 5). In contrast, piperlongumine binding weakened after pocket deletion, likely due to the absence of compensatory interactions that had initially relied heavily on aromatic stacking with the now-missing residues (Fig. 5). Overall, these findings indicate that deleting the pocket-defining residues in CCN1 does not prevent drug binding; however, it induces structural changes that promote the emergence of a new potential binding region within the C-terminal domain of the protein. This structural plasticity, along with the presence of alternative binding sites, may enable CCN1 to maintain, or even enhance, ligand interactions despite minor or substantial structural alterations.

**Fig. 6:**
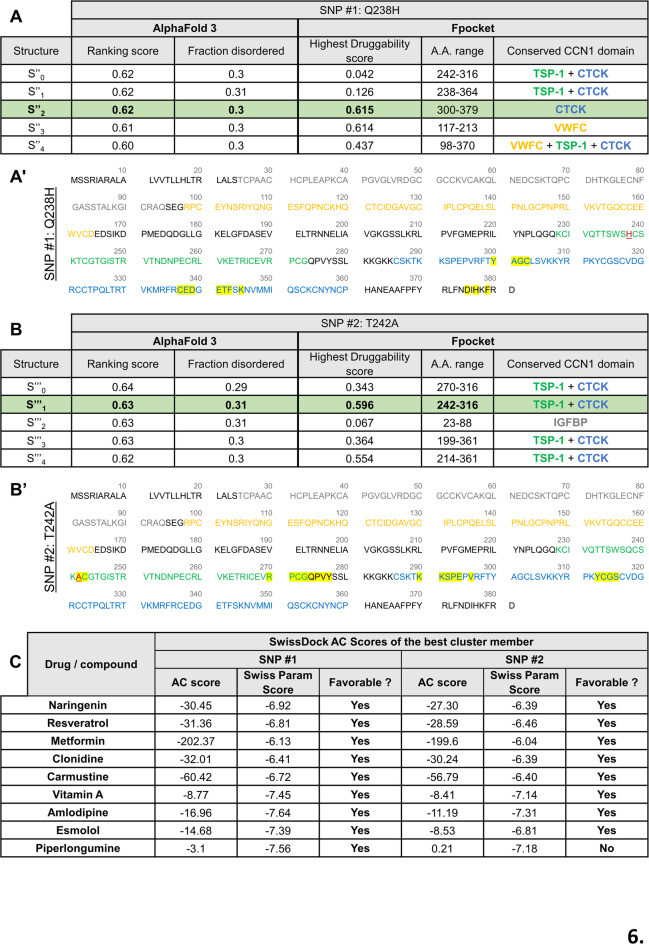
Stability of ligand-CCN1 interactions in SNP variants of CCN1. (**A**,**B**) AlphaFold 3 scores of the different predicted structures of SNP variants of CCN1 (S’’_0_-S’’_4_, for SNP1 “Q238H” and S’’’_0_-S’’’_4_, for SNP2 “T242A”). For each structure, the presence of druggable pockets were investigated using Fpocket and the a.a. forming the pocket with the highest druggability score were positioned in _*variant*_CCN1 domains. Of note, only the pocket with the highest druggability score is represented for each structure. (**A**′,**B**′) _*variant*_CCN1 sequences showing its different domains and the a.a. forming the highest predicted druggable pocket in S’’_2_ and S’’’_1_ highlighted in yellow. The different protein domains are color coded as follow: IGFBP in grey, VWFC in orange, TSP-1 in green, and CTCK in blue. Residues affected by SNPs are indicated in red and underlined in the sequence alignment. Predicted pocket in variant Q238H is mainly localized in the conserved CTCK domain, with some residues extending into the CT domain (black). In variant T242A, the pocket spans the TSP-1 and CTCK domains. (**C**) Selected compounds predicted to bind wild-type CCN1 (_*wt*_CCN1) were analyzed for their interaction with the SNP variants of CCN1 structures S’’_2_ and S’’’_1_. The key metric scores Affinity energy score (AC) and SwissParam scores are shown to predict the ligand-protein binding affinity. For each compounds different clusters are predicted, only the one with the lowest AC score (favorable) is shown.

Next, we focused on naturally occurring mutations resulting from SNPs to investigate physiologically relevant sequence variations. Specifically, we prioritized SNPs that affect amino acids most frequently predicted to interact with the ligands. The selected residues, which are affected by these SNPs, are located within the predicted druggable pocket and are consistently involved in interactions with multiple docked compounds. For example, Glutamine 238 in _*wt*_CCN1 is predicted to interact with amlodipine and piperlongumine (Fig. 2B). Substitution of Glutamine with Histidine yields a variant that remains structurally stable based on AlphaFold 3 predictions and Ramachandran plot analysis (Supplementary Fig. 1) and features a druggable pocket nested within the CTCK domain of structure S’’_2_ (Fig. 6A,A’). This variant retains the ability to interact with all nine compounds that bind _*wt*_CCN1 (Fig. 6C), indicating no deleterious consequences on CCN1 druggability. On the other hand, Threonine 242 is predicted to interact with esmolol, metformin, and naringenin (Fig. 2B). We therefore examined a naturally occurring SNP identified in population databases substituting Threonine to Alanine, and downstream analyses were conducted to evaluate its impact on CCN1-drug interactions. The AlphaFold 3–predicted models exhibited high structural stability, further supported by Ramachandran plot analysis demonstrating a robust and stereochemically reliable architecture of structure S’’’_1_ (Supplementary Fig. 1). Fpocket analysis identified a highly druggable pocket within the TSP-1/CTCK domains of this structure (Fig. 6B,B’). This pocket maintained interactions with all _*wt*_CCN1-binding ligands except for piperlongumine, which could no longer bind (Fig. 6C). These results suggest that some variants may present greater targeting challenges. They also highlight how a single amino acid substitution can cause subtle conformational shifts altering pocket accessibility or geometry and thereby affect ligand binding, particularly for compounds previously showing weak affinity to CCN1. Of note, both investigated SNP mutations did not drastically affect the 3D structure of the CCN1 (Supplementary Fig. 3), which may explain their largely preserved predicted ability to interact with the aforementioned compounds.

## Discussion

*In silico* druggability assessment emerges as a valuable tool in early drug discovery research, enabling the identification of promising protein-ligand interaction sites before pursuing costly high-throughput screening campaigns. CCN1 is a modular, secreted protein that interact with integrins, heparan sulfate proteoglycans (HSPGs), and growth factors, thereby regulating several physiological processes^1,6^. Dysregulation of CCN1 expression is often linked to several pathological conditions such as cancer, fibrosis, cardiovascular diseases, and inflammation^3^. Therefore, exploring CCN1 as a therapeutic target is appealing, especially for researchers aiming to address the diseases where CCN1 plays a pivotal role. Although CCN1 modulation has been explored using monoclonal antibodies, RNA-based approaches, or through downstream signaling pathway regulation, no small-molecule ligands have been specifically developed for this protein^1,10,12,24^. Therefore, in this study, we evaluated CCN1 druggability using structure modeling and docking analyses focusing on its interactions with drugs and bioactive compounds. Our study provides the first comprehensive *in silico* analysis of CCN1 druggability, integrating structural prediction, pocket mapping, docking, and mutational analysis. The 3D structure of CCN1 was predicted with AlphaFold 3, potential druggable pockets were identified using Fpocket, and ligand affinity was assessed with Swissdock. This multi-step computational approach offers a reliable and cost-effective strategy for early drug discovery reducing reliance on experimental screening and thereby accelerating the prioritization of candidate molecules for further validation^13,14,25^. Our findings predict the presence of a favorable druggable pocket spanning the TSP-1/CTCK domains of CCN1 located toward its C-terminus. These domains are two of the four conserved domains across all CCN family of proteins mediating their associations with integrins and most cell-ECM interactions^4,5,26^, which suggest that other CCN family members (e.g., CCN2, CCN3, etc.) may have structurally or functionally similar pockets. Interestingly, deleting all amino acids predicted to form the highest-scoring druggable pocket did not result in elimination of potential druggable regions. Instead, in this artificial multiple-point deletion mutant of CCN1, new predicted pockets emerged, primarily still within the C-terminal region, but for some also involving residues from the VWFC domain. This may likely be due to structural rearrangements and spatial folding changes resulting from the artificial amino acid deletion, possibly creating a favorable pocket interaction environment and bringing these domains into closer proximity with the C-terminus of CCN1 in its tertiary structure. Interestingly, our results show that the protein can reveal cryptic sites, which are hidden binding spots that only become accessible when the protein shifts shape. This flexibility suggests that CCN1 has more opportunities for drug binding than what static models alone might show. The observed emergence of new pockets in our results is consistent with previously reported findings in the literature. For instance, research has demonstrated that artificial mutations or deletions in proteins can uncover cryptic binding sites, which are not apparent in the unaltered protein structure. These newly exposed sites can serve as alternative binding pockets, offering new insights for drug targeting^27^. CCN1 variants arising from single nucleotide polymorphisms (SNPs) also retained druggable pockets localized toward the C-terminal region of the protein, with predicted folding comparable to wild-type CCN1. This structural conservation suggests that, despite sequence variations, the structural integrity and druggability of the C-terminal domain is preserved, potentially maintaining its functional or therapeutic relevance. Taken together, our findings indicate that the TSP-1 and CTCK domains maintain ligand-binding potential as they consistently harbor the most prominent druggable pockets within the CCN1 structure. Given their central role in CCN1-mediated cell adhesion, migration, angiogenesis, and signaling through integrins/HSPG proteoglycans, the TSP-1/CTCK domains represent stable and potentially high-value targets. Their conserved nature and structural positioning suggest they play a central role in the protein’s functional output. Therefore, identifying ligands that can bind to these domains and probably modulate CCN1’s interaction with its downstream receptors may offer promising therapeutic strategies for managing CCN1-mediated pathologies.

In structural bioinformatics, druggability refers to a protein’s capacity to bind small molecules at functionally relevant sites with high affinity and specificity, thereby modulating its biological activity^28,29^. Recent *in silico* approaches have greatly accelerated early drug discovery by enabling rapid evaluation of protein targets through structure prediction, pocket identification, and virtual docking, even without experimental structural data^13,14,25,28^. These methods also facilitate the virtual screening of natural compounds and existing drugs to identify potential binders and repurposing candidates. In fact, natural products have long been recognized as rich sources of bioactive molecules, with over 50% of FDA-approved drugs tracing their origins to natural compounds or their derivatives^30^. These molecules are valued for their structural diversity and their ability to interact with complex biological targets, positioning them as highly promising candidates in the search for novel therapeutics^31^. Several bioactive natural compounds have been shown to modulate CCN1 expression and activity, suggesting potential therapeutic benefits in CCN1-mediated disease conditions. For example, quercetin has been shown to enhance the efficacy of anticancer therapies in CCN1-overexpressing human gastric adenocarcinoma cells by targeting CCN1 and overcoming drug resistance^9^. Likewise, baicalein, another plant-derived flavonoid with antioxidant, anticancer, and anti-fibrotic properties can modulate G-protein coupled receptor and suppress fibrosis and tumor progression^10^. These findings, highlighting the therapeutic potential of targeting CCN1-mediated diseases, motivated us to screen several compounds for their binding affinity to CCN1, as direct interactions between these compounds and CCN1 have not been previously investigated. While numerous compounds influence CCN1-mediated pathways, direct targeting of CCN1, either to enhance or inhibit its receptor interactions, remains largely unexplored, highlighting the need to investigate its potential as a druggable therapeutic target. Among all tested compounds, several were predicted to interact with CCN1 and possess antioxidant, antidiabetic, senolytic, antifibrotic, chemotherapeutic, or antihypertensive properties. These findings are consistent with prior studies showing that CCN1’s C-terminal TSP-1 and CTCK domains are critical for mediating protein-protein interactions and signaling activities, including modulation of senescence, fibrosis, and angiogenesis^3^. In fact, natural compounds such as naringenin, resveratrol, and vitamin A have previously been shown to exert anti-fibrotic, antioxidant, and senolytic effects in cellular and animal models, supporting their potential relevance as CCN1 modulators^31,32^. Taken together, our findings and literature evidence collectively suggest the pivotal role of natural compounds in regulating CCN1 activity across different biological contexts. For example, quercetin, a flavonoid, effectively suppresses the senescence-associated secretory phenotype (SASP) and eliminates senescent fibroblasts, likely affecting CCN1-mediated collagen degradation in aged skin^33^. Similarly, in CCN1-overexpressing cancer cells, quercetin reduces multidrug resistance, promotes apoptosis, and enhances chemotherapeutic efficacy, consistent with a potential role of CCN1 in these processes^9^. Resveratrol promotes fibroblast migration via SIRT1-mediated upregulation of CCN1 and activation of ERK and Wnt/β-catenin signaling, highlighting the context-dependent beneficial role of CCN1 in tissue repair^34^. Retinoids, including all-trans retinoic acid and retinol, downregulate CCN1 in chronologically aged and photoaged skin, restoring type-I collagen levels and reducing MMP-1 expression, thereby improving skin structure and function^35^. Together, these studies underscore that natural compounds can regulate CCN1 expression and activity, offering possible therapeutic strategies to counteract skin aging, promote tissue repair, and overcome CCN1-associated drug resistance. Our results extend these observations by suggesting a potential molecular interaction of both natural and synthetic small molecules with the C-terminal region CCN1. This supports the concept that CCN1 can be pharmacologically modulated by structurally diverse compounds, providing a foundation for the design of CCN1-targeted therapeutics. It also expands the list beyond tested compounds to explore further ligands based on their activity and CCN1 physiological roles.

Senolytic compounds have also emerged as a promising strategy to selectively eliminate senescent cells and alleviate age-related tissue dysfunction. In models of skin aging, dasatinib combined with quercetin effectively removed senescent dermal fibroblasts, demonstrating the therapeutic potential of targeting senescence^33^. Interestingly, senescence can also play beneficial roles, as observed in hepatic stellate cells where CCN1-induced senescence is essential for proper liver regeneration. This is evidenced by impaired liver regeneration following treatment with the senolytic drug ABT263 or in a genetic mouse model^36^. Extending these observations to CCN1, our *in silico* analyses identified several compounds with senolytic or senescence-modulating properties, including piperlongumine, metformin, naringenin, and resveratrol, that can bind the most druggable C-terminal pocket of CCN1. Together, these findings support the concept that CCN1 is a valid target for senolytic intervention, and that natural and synthetic senolytic compounds could be explored to modulate CCN1 activity, selectively eliminate senescent cells, and counteract aging-related tissue dysfunction. More interestingly, our tested compounds retain their binding affinity to the multiple-point deletion mutant of CCN1 and, in fact, all exhibit even higher affinity compared to the wild type, except for piperlongumine, whose affinity slightly decreased. From a structural modeling point of view, our results suggests that CCN1 druggability is influenced by the broader structural environment, not solely by specific pocket residues. It also highlights the structural plasticity of CCN1 and the existence of alternative binding sites that, in some cases, can support ligand interaction more effectively than the original configuration. When testing the binding affinities of the selected compounds against SNP variants Q238H and T242A, no major changes were observed for most ligands, except for piperlongumine, whose binding affinity was abolished in the T242A variant. This finding is not surprising, as piperlongumine initially exhibited weak binding to _*wt*_CCN1. These results suggest that while the T242A substitution does not globally disrupt ligand binding, it may selectively hinder compounds, such as piperlongumine, that rely on subtle physicochemical features of the binding pocket. The consistent predictions across various CCN1 variants indicate that the identified interactions may be robust to genetic variation, enhancing their potential utility across a wide range of clinical and population genetic diversity. However, in some settings, our findings highlight the need to consider genetic variations in drug-target predictions, as certain variations (such as T242A substitution) may selectively impair effectiveness of specific compounds in subsets of the population. Overall, our results identify the TSP-1/CTCK domains of CCN1 as robust, conserved, and structurally adaptable druggable regions. These findings support their prioritization for small-molecule development and illustrate the utility of rapid computational approaches. Furthermore, our workflow provides a foundation for fast and reliable *in silico* testing of drug-protein interactions across diverse disease contexts and genetic backgrounds. Whether these interactions occur under physiological conditions and lead to altered CCN1-receptor interactions and downstream responses remains to be investigated through both *in vitro* and *in vivo* studies.

## Conclusion

This study provides the first computational evidence supporting CCN1 as a structurally and genetically robust druggable protein. Our integrative *in silico* workflow revealed a stable binding pocket bridging the TSP-1 and CTCK domains, capable of accommodating multiple clinically relevant compounds. The persistence of binding potential despite pocket residue deletion and natural variants highlights CCN1’s adaptable ligand-binding surface. Beyond identifying potential CCN1 modulators, this work establishes a mechanistic framework and *in silico* workflow that can accelerate the discovery of therapeutic agents targeting CCN1-mediated pathological processes such as aging, fibrosis, inflammation, and cancer.

### Limitations

This study is based on computational predictions derived from static AlphaFold models, which capture only a single conformation of CCN1 and may not represent its full dynamic behavior *in vivo*. Moreover, AlphaFold predictions do not account for post-translational modifications or the presence of ligands and cofactors, factors that can influence pocket accessibility and structural stability. In addition, Fpocket identifies binding cavities based purely on geometric features, without considering electrostatic, hydration, or allosteric effects that may affect protein-ligand interactions. Finally, although our in silico findings suggest potential CCN1-ligand interactions, experimental validation remains essential to confirm their biological relevance and therapeutic significance.

## Supplementary Information

Below is the link to the electronic supplementary material.

Supplementary Material 1

Supplementary Material 2

## Data Availability

All data supporting the findings of this study are included in the supplementary file accompanying this article.
