# Supplementary material for: Computational analysis of CCN1 as a druggable target predicts interactions with bioactive compounds
Source: Sci Rep. 2026 Jan 13;16:4039. doi: 10.1038/s41598-025-34139-4 (PMC12855203; doi:10.1038/s41598-025-34139-4)
Supplement: Supplementary file 2 — Supplementary Material 2 [file 41598_2025_34139_MOESM2_ESM.docx]

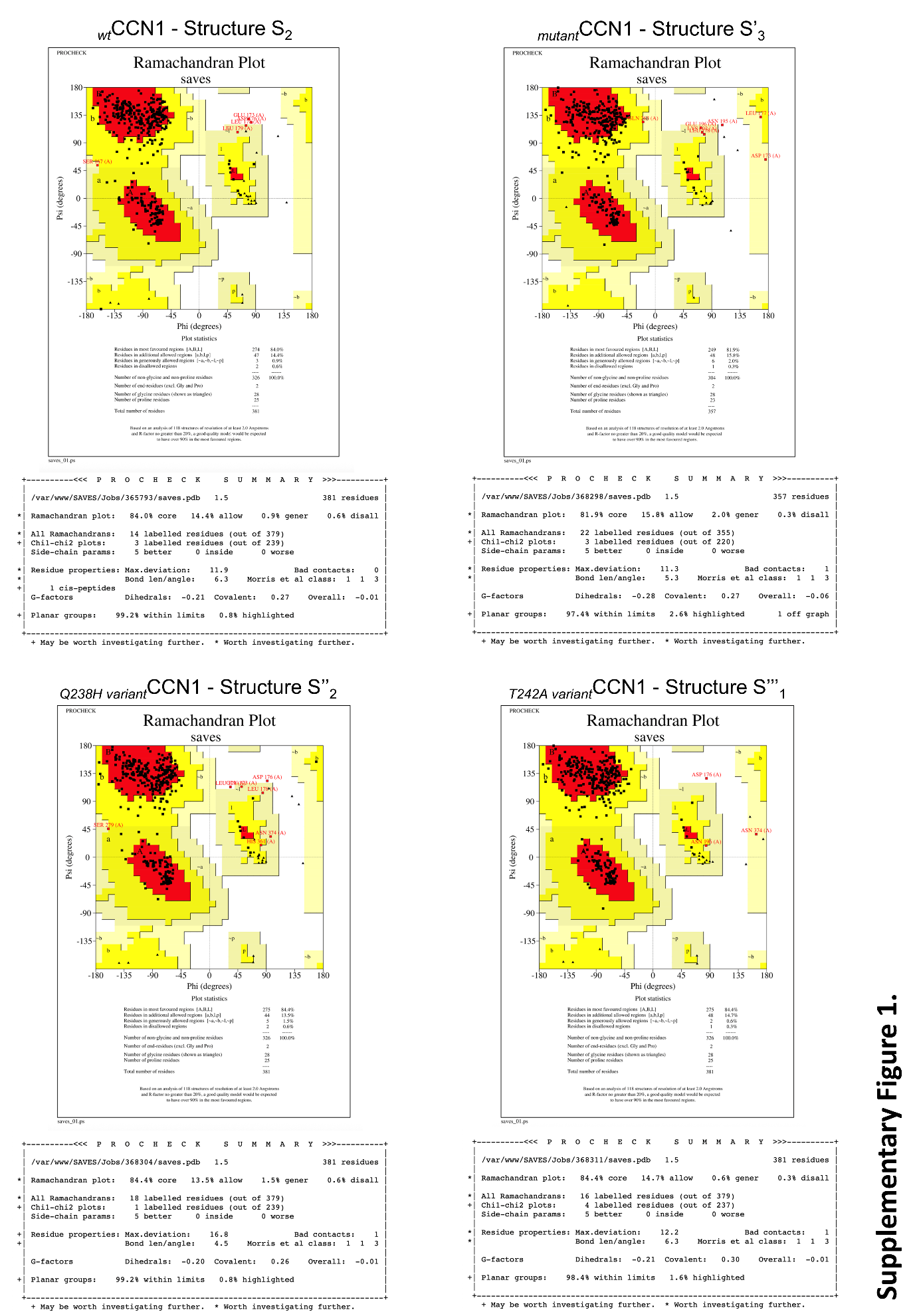


**Supplementary Figure 1. PROCHECK analysis of the different CCN1 structures used in this study.** For each structure, the Ramachandran plot is shown, displaying residues (black) located in the most favored regions (red), additionally allowed regions (yellow), generously allowed regions (light yellow), and disallowed regions (white), along with the corresponding PROCHECK summary.

**
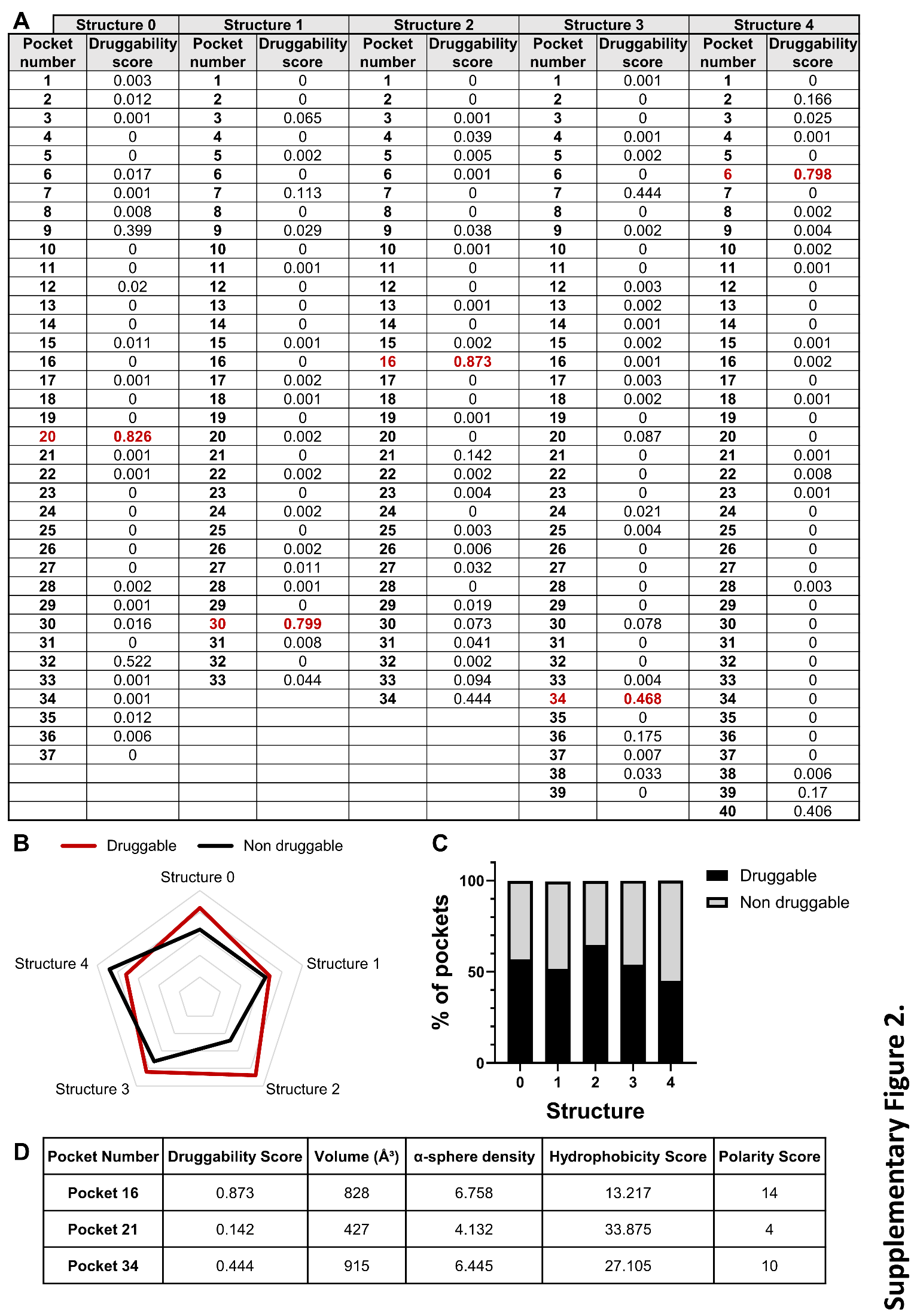
**

**Supplementary Figure 2. Detailed results of druggable pocket predictions using Fpocket. (A)** For each structure predicted by AlphaFold, the total number of pockets is shown along with their respective druggability scores. Pockets highlighted in red represent those with the highest druggability scores and are also shown in Figure 1A. Spider chart **(B)** and tacked histogram **(C)** comparing the proportion of druggable pockets across the different structures (S_0_–S_4_). Structure Two (S_2_) shows the highest percentage of druggable pockets and the highest druggability score of one of its pockets, suggesting a greater potential for targeted drug binding compared to the other structures analyzed. **(D)** Summary of the highest-scoring pockets in the CCN1 S2 model, including computed physicochemical parameters reflecting their stability and hydropathic nature.


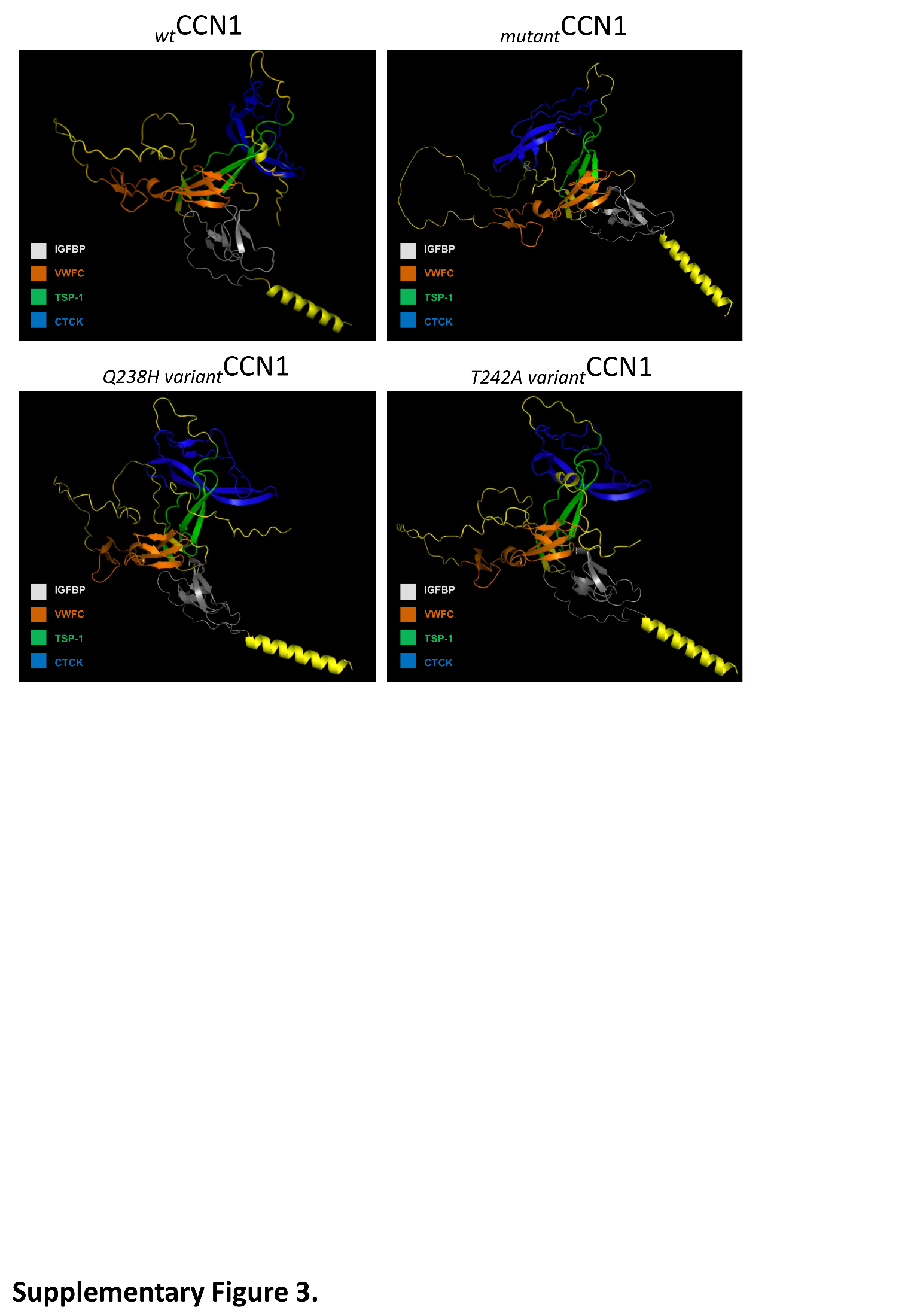


**Supplementary Figure 3.** 3D structural visualization of wild-type CCN1 (*_wt_*CCN1), the artificial multiple-point deletion mutant, and the Q238H and T242A SNP variants, as predicted by AlphaFold 3 and visualized using PyMOL. Moderate structural changes were observed in the mutant, while the variants exhibited slight alterations compared to the wild type.
